# Examination of the Anaerobic Growth of *Campylobacter concisus* Strains

**DOI:** 10.1155/2014/476047

**Published:** 2014-08-20

**Authors:** Hoyul Lee, Rena Ma, Michael C. Grimm, Stephen M. Riordan, Ruiting Lan, Ling Zhong, Mark Raftery, Li Zhang

**Affiliations:** ^1^The School of Biotechnology and Biomolecular Sciences, University of New South Wales, Kensington, Sydney, NSW 2052, Australia; ^2^St George and Sutherland Clinical School, University of New South Wales, Sydney, NSW 2052, Australia; ^3^Gastrointestinal and Liver Unit, Prince of Wales Hospital and Prince of Wales Clinical School, University of New South Wales, Sydney, NSW 2052, Australia; ^4^Bioanalytical Mass Spectrometry Facility, University of New South Wales, Sydney, NSW 2052, Australia

## Abstract

*Campylobacter concisus* is an oral bacterium that is associated with intestinal diseases. *C. concisus* was previously described as a bacterium that requires H_2_-enriched microaerobic conditions for growth. The level of H_2_ in the oral cavity is extremely low, suggesting that *C. concisus* is unlikely to have a microaerobic growth there. In this study, the anaerobic growth of *C. concisus* was investigated. The growth of fifty-seven oral *C. concisus* strains and six enteric *C. concisus* strains under various atmospheric conditions including anaerobic conditions with and without H_2_ was examined. The atmospheric conditions were generated using commercially available gas-generation systems. *C. concisus* putative virulence proteins were identified using mass spectrometry analysis. Under anaerobic conditions, 92% of the oral *C. concisus* strains (52/57) and all six enteric strains grew without the presence of H_2_ and the presence of H_2_ greatly increased *C. concisus* growth. An oral *C. concisus* strain was found to express a number of putative virulence proteins and the expression levels of these proteins were not affected by H_2_. The levels of H_2_ appeared to affect the optimal growth of *C. concisus*. This study provides useful information in understanding the natural colonization site and pathogenicity of *C. concisus*.

## 1. Introduction

*Campylobacter concisus* is a Gram-negative bacterium that is commonly present in the human oral cavity [1, 2]. In some individuals,* C. concisus* may colonize the intestinal tract and was found to be associated with inflammatory bowel disease (IBD) due to its significantly higher prevalence in the intestinal tract of patients with IBD as compared to controls [3–6]. IBD is a chronic inflammatory disease of the gastrointestinal tract (GIT) with unknown aetiology. Crohn's disease (CD) and ulcerative colitis (UC) are the two major clinical forms of IBD [7]. In addition to IBD,* C. concisus* was also often isolated from diarrheal stool samples, suggesting a possible involvement in diarrheal disease [8–10].

In the literature, it was described that* C. concisus* requires H_2_-enriched microaerobic conditions for growth [11, 12]. In laboratory cultivations of* C. concisus,* microaerobic conditions enriched with 5–10% H_2_ have been used [2, 9, 12, 13]. The primary colonization site of* C. concisus* is the human oral cavity [1, 2]. The level of H_2_ in the human oral cavity is extremely low [14]. Given this, it is unlikely that* C. concisus* is able to grow microaerobically in the human oral cavity. In previous studies,* C. concisus* was isolated from gingival plaque and saliva, locations where a large number of anaerobes were found, suggesting that* C. concisus* is more likely to use an anaerobic growth in the human oral cavity [1, 2, 15].

To date, there have been no studies that systematically examined the growth of* C. concisus* under anaerobic conditions. Furthermore, there is no information available regarding the impact of H_2_ on* C. concisus* growth under anaerobic conditions. These issues were investigated in the current study. Furthermore, we examined the expression of putative virulence proteins of an oral* C. concisus* strain grown under anaerobic conditions.

## 2. Materials and Methods

### 2.1. *C. concisus* Strains Used in This Study

A total of 63* C. concisus* strains were examined in this study, including 57 oral* C. concisus* strains and six enteric strains. Of the 57 oral* C. concisus* strains, 19 strains were from patients with CD, 14 strains from patients with UC, and 24 strains from healthy individuals. Of the six enteric* C. concisus* strains, five strains were isolated from patients with IBD including two strains isolated from intestinal biopsies of patients with UC, two strains isolated from fecal samples of patients with UC, and one strain isolated from intestinal biopsies of a patient with CD. Both oral and enteric* C. concisus* strains were isolated in our previous studies [1, 3, 6].* C. concisus* strain 13826, which was isolated from fecal samples of a patient with bloody diarrhea, was purchased from the American Type Culture Collection.

### 2.2. Examination of* C. concisus* Growth under Various Atmospheric Conditions

The growth of the above 63* C. concisus* strains under the following atmospheric conditions was examined.

#### 2.2.1. Anaerobic Condition without H_2_ (Anaero^H2−^)

Anaero^H2−^ condition was generated using AN25A gas-generation system as instructed by the manufacturer (Oxoid, Hampshire, UK).

#### 2.2.2. Anaerobic Condition Containing 9% of H_2_ (Anaero^H2+^)

Anaero^H2+^ condition was generated using BR38B gas-generation system, which was placed into a 3.5 L jar in the presence of a catalyst following the manufacturer's instruction (Oxoid).

#### 2.2.3. Microaerobic Condition without H_2_ (Micro^H2−^)

A Micro^H2−^ condition was generated using two different gas-generation systems, following the manufacturer's instruction (Oxoid). The first gas-generation system was BR56A (Micro^H2−a^), which was placed into a 3.5 L jar in the presence a catalyst. The second gas-generation system was CN25A (Micro^H2−b^), which was placed into a 2.5 L jar.

#### 2.2.4. Original Isolation Condition

The* C. concisus* strains used in this study were isolated in our previous studies [1, 3, 6]. The atmospheric condition used in isolation of these* C. concisus* strains from clinical samples in our previous studies was to place the BR56A gas-generation system into a 2.5 L jar with a catalyst, which was a modification of the manufacturer's instruction. In this study, we refer to this atmospheric condition as the original isolation condition (Ori^con^).

Each of the 63* C. concisus* strains was streaked onto three horse blood agar (HBA) plates for each atmospheric condition. The HBA plates were prepared using blood agar base number 2 supplemented with 6% (v/v) heat-inactivated defibrinated horse blood and 10 *μ*g/mL of vancomycin (Oxoid). Following incubation at 37°C under Anaero^H2−^, Anaero^H2+^, Micro^H2−a^, Micro^H2−b^, and Ori^con^ conditions, respectively, for 48 hours, plates were examined for the appearance of colonies under a stereo microscope. The morphology of all grown* C. concisus* strains was examined using a phase-contrast microscope.

### 2.3. Quantitative Comparison of* C. concisus* Growth under Anaero^H2−^ and Anaero^H2+^ Conditions

To further quantitatively compare the growth of* C. concisus* under Anaero^H2−^ and Anaero^H2+^ conditions, the colony forming unit (CFU) of 12* C. concisus* strains grown under these two conditions was determined. These 12 strains included six oral strains from patients with CD, three oral strains from UC, two oral strains from healthy controls, and one enteric strain from a patient with UC. These 12 strains were randomly selected from the 63 strains used in the experiments in Section 2.2.

*C. concisus* strains were first cultured on HBA plates under Ori^con^ condition at 37°C for 48 hours. The bacterial cells were collected and washed once with phosphate buffered saline (PBS). The bacterial pellet of each strain was resuspended in PBS and OD_600_ was adjusted to 0.05, which was used as the initial inoculum for further assessment of the growth of* C. concisus* under Anaero^H2−^ and Anaero^H2+^ conditions.

The initial inoculum suspension (50 *μ*L) of each* C. concisus* strain was inoculated onto six HBA plates using a sterile L-shaped glass rod. Three plates were incubated under Anaero^H2−^ condition and the remaining three plates were incubated under Anaero^H2+^ condition for 48 hours at 37°C.

The bacterial cells of each* C. concisus* strain collected from the three plates incubated under Anaero^H2−^ condition and the three plates incubated under Anaero^H2+^ condition were pooled, respectively (1 mL of PBS was used for collection of bacterial cells from each plate). From each pooled* C. concisus* suspension, serial dilutions (1 : 10 to 1 : 10^8^) were prepared. Each of the dilutions (5 *μ*L) was inoculated onto HBA plates in triplicate. The plates were further incubated under Ori^con^ condition for 48 hours at 37°C to determine the CFU numbers.

### 2.4. Examination of* C. concisus* Growth under Anaerobic and Microaerobic Conditions in the Presence of Different Concentrations of H_2_

P3UCO-S1 strain was used for this experiment. P3UCO-S1 strain is an oral strain previously isolated from a patient with UC [6, 16]. In a previous study of analysis of six housekeeping genes, we showed that the six housekeeping genes of P3UCO-S1 strain were identical to the strain isolated from intestinal biopsies of the same patient (P3UCB-S1) suggesting that this oral* C. concisus* strain was able to colonize the intestinal tract. Given this, we have decided to use P3UCO-S1 strain in this part of the study.

P3UCO-S1 strain was first cultured on a HBA plate under Ori^con^ condition for 48 hours at 37°C. Following this, bacterial cells were collected and suspended into PBS and the OD_600_ was adjusted to 0.05. The bacterial suspension (30 *μ*L) was inoculated onto 18 HBA plates. The plates were incubated under either anaerobic conditions or microaerobic conditions containing various conditions of H_2_ (three plates in each condition). Anaerobic and microaerobic conditions were generated using gas-generation system AN25A and gas-generation system CN25A, respectively (Oxoid). Hydrogen gas was supplemented by including 0.021 g, 0.042 g, or 0.083 g of sodium borohydride and 10 mL of H_2_O in a container placed in a 2.5 L jar, respectively, which generated 2.5%, 5.0%, and 10.0% of H_2_, respectively. H_2_ gas was generated by the chemical reaction NaBH_4_ + 4H_2_O = 4H_2_ + NaB(OH)_4_.

After a period of 48 hours of incubation at 37°C,* C. concisus* bacterial cells were collected from each plate using 1 mL of PBS. Three plates cultured under each condition were pooled and eight serial dilutions (1 : 10 to 1 : 10^8^) were prepared. Each of the eight dilutions (5 *μ*L) was inoculated onto HBA plates in four replicates. The plates were further incubated under Ori^con^ condition for 48 hours at 37°C to determine the CFU numbers.

### 2.5. Putative Virulence Proteins Expressed by an Oral* C. concisus* Strain Cultured under Anaero^H2−^ and Anaero^H2+^ Conditions

Proteins expressed by* C. concisus* cultured under Anaero^H2−^ and Anaero^H2+^ conditions were analysed using mass spectrometry.* C. concisus* strain P6CDO-S1 was used in this experiment. P6CDO-S1 is an oral strain previously isolated from saliva of a patient with CD. Our previous studies showed that this oral* C. concisus* strain was genetically close to a* C. concisus* strain isolated from the intestinal biopsies of a patient with CD [6]. Therefore, we decided to investigate whether this oral* C. concisus* strain expresses putative virulence proteins and whether these proteins are expressed differentially when the strain is grown under Anaero^H2−^ and Anaero^H2+^ conditions.

Briefly,* C. concisus* P6CDO-S1 strain was grown on HBA plates for 48 hours under Anaero^H2−^ and Anaero^H2+^ conditions, respectively.* C. concisus* bacteria were collected and washed with PBS and then 19 *μ*g whole cell proteins were separated on 12% SDS-PAGE as described previously [16]. The gel lane of each sample was cut into 10 slices. In-gel protein trypsin digestion was performed. The extracted peptides were separated by liquid chromatography and analysed by MS/MS as previously described [16, 17].

Mascot Daemon program (Matrix Science, London, UK) was used for bacterial protein identification against the NCBI database. The spectral counts of the same proteins expressed by P6CDO-S1 under Anaero^H2−^ and Anaero^H2+^ conditions were compared using the Scaffold-3 software (Proteome software, OR, USA) [18]. The experiment was carried out in duplicate and repeated twice.

Mass spectrometry was conducted at the Bioanalytical Mass Spectrometry Facility, University of New South Wales, Australia.

### 2.6. Statistical Analysis

Unpaired *t*-test was used for comparison of CFU numbers. Fisher's exact test was used for analysis of the growth rate of* C. concisus* strains isolated from patients with IBD and controls. GraphPad Prism 5 software was used for statistical analysis (San Diego, CA). *P* value < 0.05 was considered a significant difference.

## 3. Results

### 3.1. The Growth of* C. concisus* Strains under Different Atmospheric Conditions

Of the 57 oral strains examined, 52 strains (91%) grew under Anaero^H2−^ conditions. Of the oral* C. concisus* strains isolated from patients with CD and UC, the positive growth rates under Anaero^H2−^ condition were 84% (16/19) and 86% (12/14), respectively, which were not statistically different from the positive growth rate of oral* C. concisus* strains (100%, 24/24) isolated from healthy controls (*P* > 0.05) (Table 1). All oral* C. concisus* strains grew under Anaero^H2+^ conditions (Table 1).

All six enteric* C. concisus* strains grew under both Anaero^H2−^ and Anaero^H2+^ conditions.

The colonies of* C. concisus* strains, both the oral and enteric strains, grown under Anaero^H2−^ conditions appeared much smaller than those grown under Anaero^H2+^ conditions. The morphology of* C. concisus* grown under Anaero^H2−^ and Anaero^H2+^ conditions was not different under phase contrast microscopy.

None of the* C. concisus* strains grew under microaerobic condition without H_2_; no bacterial colonies were observed on plates cultured under both Micro^H2−a^ and Micro^H2−b^ conditions. All strains grew under Ori^con^ condition.

### 3.2. Quantitative Comparison of* C. concisus* Growth under Anaero^H2−^ and Anaero^H2+^ Conditions

To further compare the growth of* C. concisus* strains under Anaero^H2+^ and Anaero^H2−^ conditions, the CFUs of 12* C. concisus* strains grown under these two atmospheric conditions were determined. All strains had a greatly increased growth under Anaero^H2+^ condition in comparison to the Anaero^H2−^ condition. The CFU numbers of all 12* C. concisus* strains grown under Anaero^H2+^ condition were significantly higher than those of the respective strains grown under Anaero^H2−^ condition (*P* < 0.05) (Table 2).

### 3.3. The Growth of* C. concisus* under Anaerobic and Microaerobic Conditions Containing Different Concentrations of H_2_

**Figure 1:**
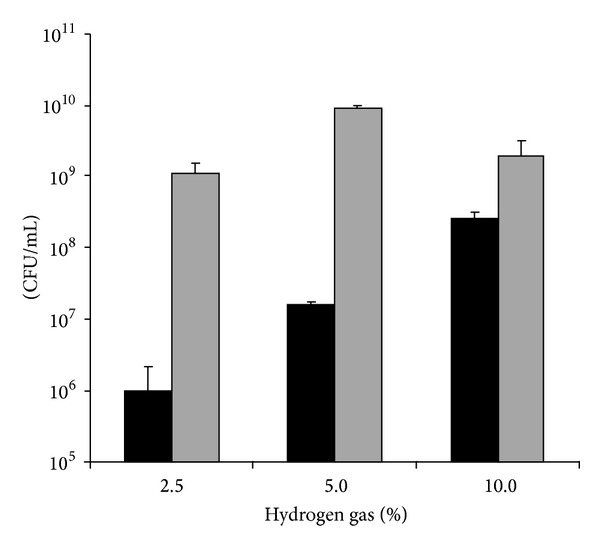
Growth of* C. concisus* P3UCO-S1 strain under various hydrogen gas concentrations. CFU of* C. concisus* was measured after incubation under microaerobic (black bar) or anaerobic (grey bar) condition with different hydrogen gas concentrations for 48 hours. The experiment was repeated twice. The error bar indicates the standard deviation. P3UCO-S1 strain is an oral strain isolated from a patient with UC.

P3UCO-S1 strain was used as a representative strain to evaluate the growth of* C. concisus* under anaerobic and microaerobic conditions containing different concentrations of H_2_. Under anaerobic conditions, the CFUs of P3UCO-S1 strain cultured in the presence of 2.5%, 5%, and 10% H_2_ were (1.10 ± 0.42) × 10^9^/mL, (9.15 ± 0.82) × 10^9^/mL, and (1.90 ± 1.33) × 10^9^/mL, respectively. The CFU numbers of 5% H_2_ were significantly higher than the CFU number of 2.5% and 10% H_2_ (both *P* < 0.0001). The CFU numbers of 10% H_2_ and 2.5% H_2_ were not significantly different (*P* = 0.3) (Figure 1).

Under microaerobic conditions, the CFU numbers of P3UCO-S1 strain cultured in the presence of 2.5%, 5%, and 10% H_2_ were (1.0 ± 1.15) × 10^6^/mL, (1.60 ± 0.16) × 10^7^/mL, and (2.67 ± 0.5) × 10^8^/mL, respectively. The CFU number of 2.5% H_2_ was significantly lower than the CFU numbers of 5% and 10% H_2_ (*P* < 0.0001 and *P* < 0.005, resp.). The CFU number of 5% H_2_ was significantly lower than the CFU number of 10% H_2_ (*P* < 0.005) (Figure 1).

### 3.4. Putative Virulence Proteins Expressed by* C. concisus* P6CDO-S1 Strain Cultured under Anaero^H2−^ and Anaero^H2+^ Conditions

Proteins expressed by strain P6CDO-S1 under Anaero^H2+^ and Anaero^H2−^ conditions were subjected to mass spectrometry analysis. A number of putative virulence proteins such as fibronectin-binding protein, outer membrane protein (Omp), protease htpx, S-layer-RTX protein, hemagglutinin/hemolysin-related protein, CjaC, and EvpB family type VI secretion protein were identified. The expression levels of these putative virulence proteins, indicated by the spectral counts, were not statistically different when* C. concisus* strain P6CDO-S1 was grown under Anaero^H2+^ and Anaero^H2−^ conditions (Table 3).

## 4. Discussion

In this study, the growth of* C. concisus* strains under different atmospheric conditions was examined. It was previously described that* C. concisus* is a bacterium which requires H_2_-enriched microaerobic conditions for growth and some* C. concisus* strains may grow under anaerobic conditions if fumarate and formate are present in the culture plates [2, 11, 12]. In this study, we found that under anaerobic conditions the majority of oral* C. concisus* strains (91%, 52/57) grew on HBA plates containing no formate or fumarate without the presence of H_2_, suggesting that oral* C. concisus* is an anaerobic bacterium and that H_2_ gas, formate, and fumarate are not essential requirements for the anaerobic growth of oral* C. concisus* strains. None of the 57 oral* C. concisus* strains grew under microaerobic conditions without H_2_, suggesting that microaerobic growth of* C. concisus* requires the presence of H_2_, which is consistent with previous findings [9, 11].

Under anaerobic conditions, the presence of H_2_ greatly increased the growth of* C. concisus*, demonstrated by the increased colony sizes observed macroscopically and the increased CFU numbers of the same strain cultured under Anaero^H2+^ and Anaero^H2−^ conditions (Table 2). These results suggest that under anaerobic conditions* C. concisus* has different metabolic pathways in generating energy for growth and oxidization of H_2_ is a pathway generating high energy for a rapid growth. The solubility of H_2_ gas in H_2_O is extremely low; thus liquid culture methods are not suitable for assessing the impact of H_2_ gas on* C. concisus* growth [19]. Given this, in this study, the CFU numbers of* C. concisus* strains were determined using a plate culture method.

In humans, H_2_ is produced by anaerobic bacteria predominantly in the colon [20, 21]. H_2_ generated in the intestine is disposed by H_2_ consuming bacteria such as methanogenic bacteria, sulfate-reducing bacteria, and acetogens [22]. Some H_2_ is diffused into blood and this H_2_ can be measured by breath testing [23]. Dietary factors and the composition of an individual's intestinal microbiota affect intestinal H_2_ production and consumption [24–26]. The natural host of* C. concisus* is humans and the primary colonization site is the human oral cavity [1, 2]. The concentration of excreted H_2_ in the oral cavity is extremely low. The basal level of hydrogen in healthy individuals is usually less than 10 ppm, thus having a H_2_ level of less than 0.001% (1 ppm = 0.0001%) [14]. In addition to anaerobic bacteria in the intestine, oral anaerobic bacteria may also produce H_2_ by fermentation of carbohydrate residues from food. However, the level of H_2_ produced by oral anaerobes is very low. Mastropaolo and Rees showed that, following a solid meal, the H_2_ produced by oral anaerobes was 25 ppm (0.0025%) and this level was retained for only 73 minutes [27]. Given this,* C. concisus* colonizing the oral cavity is unlikely to have constantly available H_2_ for growth. The finding in this study that oral* C. concisus* strains were able to grow without the presence of H_2_ under anaerobic conditions helps to explain why* C. concisus* is able to colonize the human oral cavity.

Despite the fact that H_2_ dramatically increases the growth of* C. concisus* and the intestine is the dominant place for H_2_ production in humans, it is interesting to note that* C. concisus* has selected the oral cavity, rather than the intestinal environment, as its natural colonization site. This suggests that in healthy individuals there are some factors in the gastrointestinal tract that inhibit* C. concisus* intestinal colonization. It is likely that such inhibitory factors are low or lacking in patients with IBD, which contributes to the higher intestinal prevalence of* C. concisus* in these patients. One of such factors may be methanogenic bacteria, the dominant H_2_ consuming bacteria in the human intestine that produce methane. It is possible that methanogenic bacteria in the intestine compete with* C. concisus* for use of H_2_.

A study by McKay et al. examining hydrogen and methane excretion in patients with IBD and controls showed that the prevalence of methane excretion was 13% in patients with CD and 15% in patients with UC, which was significantly lower than that in healthy controls (54%) [28]. This observation was supported by a study from Pimentel et al., which showed that 97% of patients with IBD (75/78), who had predominantly a diarrheal condition, excreted H_2_ only and no methane [29]. These results suggest that there is a low level of methanogenic bacteria in patients with IBD. Indeed, a study conducted by Scanlan et al. detected a low prevalence of intestinal methanogenic bacteria in patients with IBD in comparison to healthy controls and other disease groups [30]. Methanogenic bacteria play a predominant role in disposing intestinal H_2_ in humans [31]. The lack of sufficient intestinal methanogenic bacteria in patients with IBD may have generated an intestinal environment that allows* C. concisus* to use H_2_ for a rapid growth.

We previously showed that some oral* C. concisus* strains were able to colonize the intestinal tract and have the potential to cause enteric disease [16, 32]. In this study, we found that P6CDO-S1 strain, an oral* C. concisus* strain isolated from a patient with CD, expressed a number of putative virulence proteins. These proteins were previously reported to contribute to the virulence of other bacterial species [32–39]. However, their roles in* C. concisus* virulence remain to be characterized. If indeed these putative proteins play a role in* C. concisus* virulence, the finding in this study that the expression levels of these proteins remain similar when P6CDO-S1 strain is cultured under anaerobic conditions with and without H_2_ suggests that the impact of H_2_ on* C. concisus* virulence is unlikely through affecting these proteins. It is likely that H_2_ may affect* C. concisus* virulence through increasing the growth of* C. concisus* to a disease-causing threshold.

This study also found that, under anaerobic conditions, P3UCO-S1 strain, an oral strain isolated from a patient with UC, had a significantly higher CFU in the presence of 5% H_2_, as compared to 2.5% H_2_ and 10% H_2_. Under microaerobic conditions, this strain had a significantly higher CFU in the presence of 10% H_2_ compared to 2.5% and 5% H_2_. It appeared that the concentrations of H_2_ supplied in bacterial cultivation affect the optimal growth of* C. concisus*. This aspect should be further investigated by examining more* C. concisus* strains using systems that are able to supply fixed concentrations of CO_2_, N_2_, and H_2_, which will provide useful information to clinical laboratories in isolation of* C. concisus* from clinical samples.

In addition to the 57 oral* C. concisus* strains, we have included six enteric strains, with five strains being isolated from patients with IBD, into this study. These enteric strains showed an anaerobic growth pattern that was similar to oral* C. concisus* strains.

In summary, this study found that oral* C. concisus* strains were able to grow under anaerobic conditions without the presence of H_2_, formate, or fumarate and that these strains did not grow in microaerobic conditions without H_2_, suggesting that they are anaerobes. The presence of H_2_ in the anaerobic conditions greatly increased the growth of oral* C. concisus* strains. Using mass spectrometry analysis, an oral* C. concisus* strain isolated from a patient with CD was found to express a number of putative virulence proteins and the expression levels of these proteins under anaerobic conditions with and without H_2_ remained similar. While the numbers of enteric* C. concisus* strains included in this study were small, these enteric strains and oral* C. concisus* had a similar anaerobic growth pattern. This study provides useful information in understanding the natural colonization site and pathogenicity of* C. concisus*.

## Figures and Tables

**Table 1:** Positive growth rates of oral *C. concisus* strains under Anaero^H2−^ and Anaero^H2+^ conditions.

Strains	Anaero^H2−^	Anaero^H2+^
Strains from CD (*n* = 19)	84%	100%
Strains from UC (*n* = 14)	86%	100%
Strains from control (*n* = 24)	100%	100%

Total strains (*n* = 57)	91%	100%

Anaero^H2−^: anaerobic conditions without H_2_.

Anaero^H2+^: anaerobic conditions with H_2_.

All strains did not grow under microaerobic conditions without H_2_ (Micro^H2−a^ and Micro^H2−b^).

**Table 2:** CFU of *C. concisus* strains cultured under Anaero^H2+^ and Anaero^H2−^ conditions.

Strain	Anaero^H2+^ CFU	Anaero^H2−^ CFU
P1CDO-S1	517 ± 23.6	0.863 ± 0.061
P1CDO-S2	328 ± 25.5	0.813 ± 0.0318
P2CDO-S1	93.0 ± 8.49	1.36 ± 0.212
P4CDO-S1	123 ± 4.71	0.195 ± 0.0252
P6CDO-S1	126 ± 9.55	1.21 ± 0.125
P8CDO-S1	112 ± 14.4	3.15 ± 0.388
P3UCO-S1	550 ± 70.7	1.43 ± 0.0106
P7UCO-S1	311 ± 105	9.42 ± 0.118
P13UCO-S3	111 ± 2.12	6.50 ± 0.707
P3UCB-S1	146 ± 36.8	1.57 ± 0.330
H3O-S1	73.8 ± 15.9	9.50 ± 1.91
H5O-S1	135 ± 5.30	9.50 ± 0.707

The values (means ± standard deviation) were from triplicates. CFU: (colony forming unit) × 10^8^/mL. The CFU numbers of all 12 strains grown under Anaero^H2+^ conditions were significantly higher than those of the respective strains grown under Anaero^H2−^ conditions (*P* < 0.05). Strains P1CDO-S1, P1CDO-S1, P2CDO-S1, P4CDO-S1, P6CDO-S1, and P8CDO-S1 were oral strains isolated from patients with CD. Strains P3UCO-S1, P7UCO-S1, and P13UCO-S3 were oral strains isolated from patients with UC. Strain P3UCB-S1 was an enteric strain isolated from intestinal biopsies of a patient with UC. Strains H3O-S1 and H5O-S1 were oral strains isolated from healthy controls.

**Table 3:** Putative virulence proteins expressed by *C. concisus* P6CDO-S1 strain cultured under Anaero^H2+^ and Anaero^H2−^ conditions∗.

Proteins	Locus tag	SC (Anaero^H2+^)	SC (Anaero^H2−^)
Flagellin B	CCC13826_2297	16.9 ± 3.59	15.1 ± 3.96
Fibronectin-binding protein	CCC13826_0739	12.8 ± 3.43	12.8 ± 1.98
Protease htpx	CCC13826_1039	8.44 ± 0.990	10.5 ± 1.93
Omp18	CCC13826_0923	7.49 ± 4.48	7.80 ± 3.51
S-layer-RTX protein	CCC13826_1838	8.15 ± 3.74	4.00 ± 1.62
CjaC	CCC13826_0963	4.93 ± 2.90	6.33 ± 0.768
EvpB family type VI secretion protein	CCC13826_1182	4.96 ± 0.555	5.33 ± 0.722
Hemagglutinin/hemolysin-related protein	CCC13826_0009	4.39 ± 1.77	2.33 ± 0.667

∗Putative virulence proteins were identified using mass spectrometry analysis.

SC: the value of the mean spectral counts from four replicates with standard deviation. The SC values of virulence proteins in *C. concisus* P6CDO-S1 strain cultured under Anaero^H2+^ and Anaero^H2−^ conditions were not significantly different (*P* > 0.05).

Anaero^H2+^: anaerobic condition with H_2_.

Anaero^H2−^: anaerobic condition without H_2_.

P6CDO-S1 strain is an oral strain isolated from a patient with CD.
